# Supplementary figures and images for: Activation of the tick Toll pathway to control infection of Ixodes ricinus by the apicomplexan parasite Babesia microti
Source: PLoS Pathog. 2024 Dec 16;20(12):e1012743. doi: 10.1371/journal.ppat.1012743 (PMC11649134; doi:10.1371/journal.ppat.1012743)

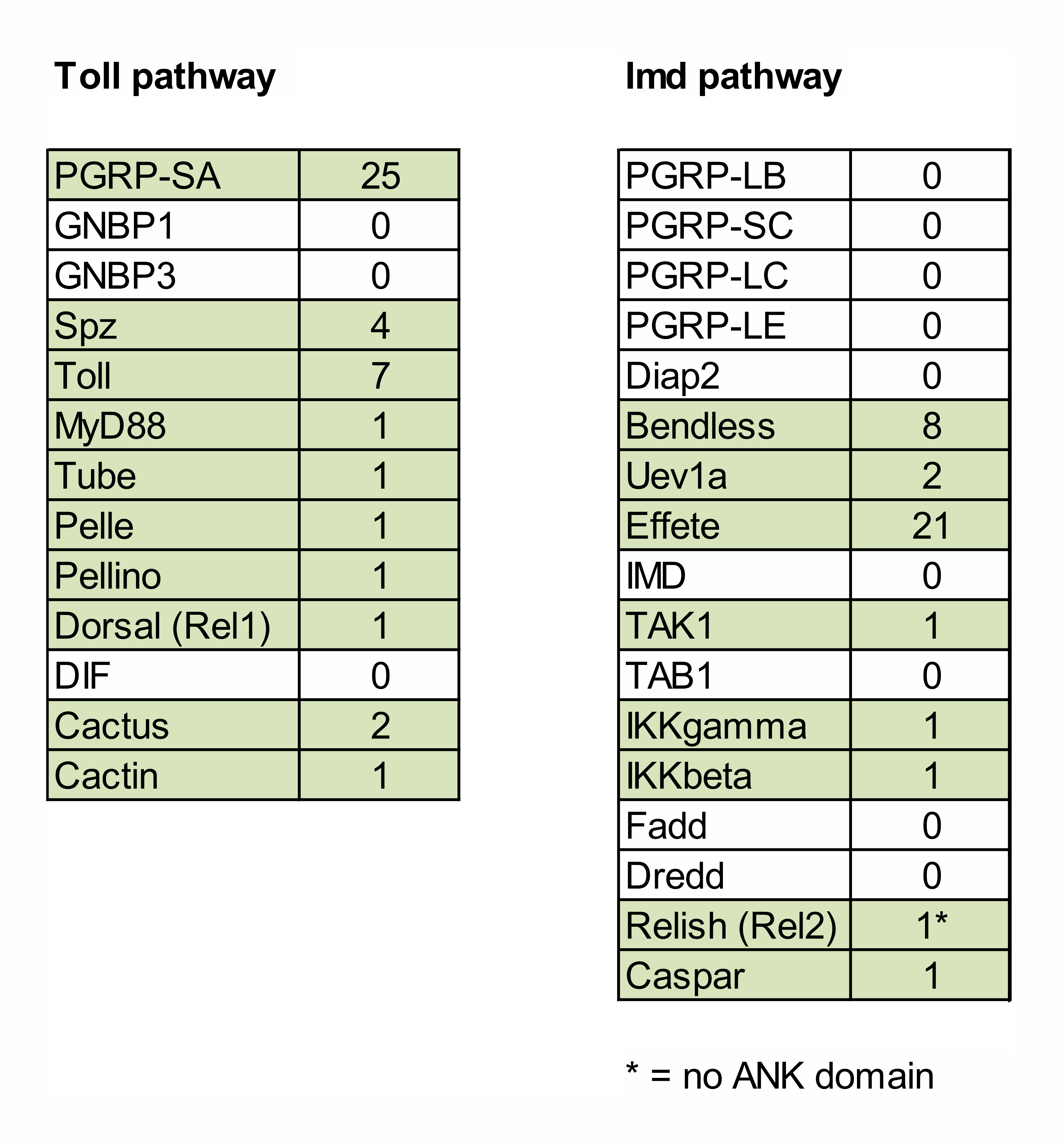

Supplement: S1 Fig — In silico screening of the nymphal cDNA database of I. ricinus (Bioproject PRJNA657487) for components of the Toll and IMD pathways. ANK = ankyrin. Details of the screening can be found in S1 Data. (TIF) [file ppat.1012743.s001.tif]

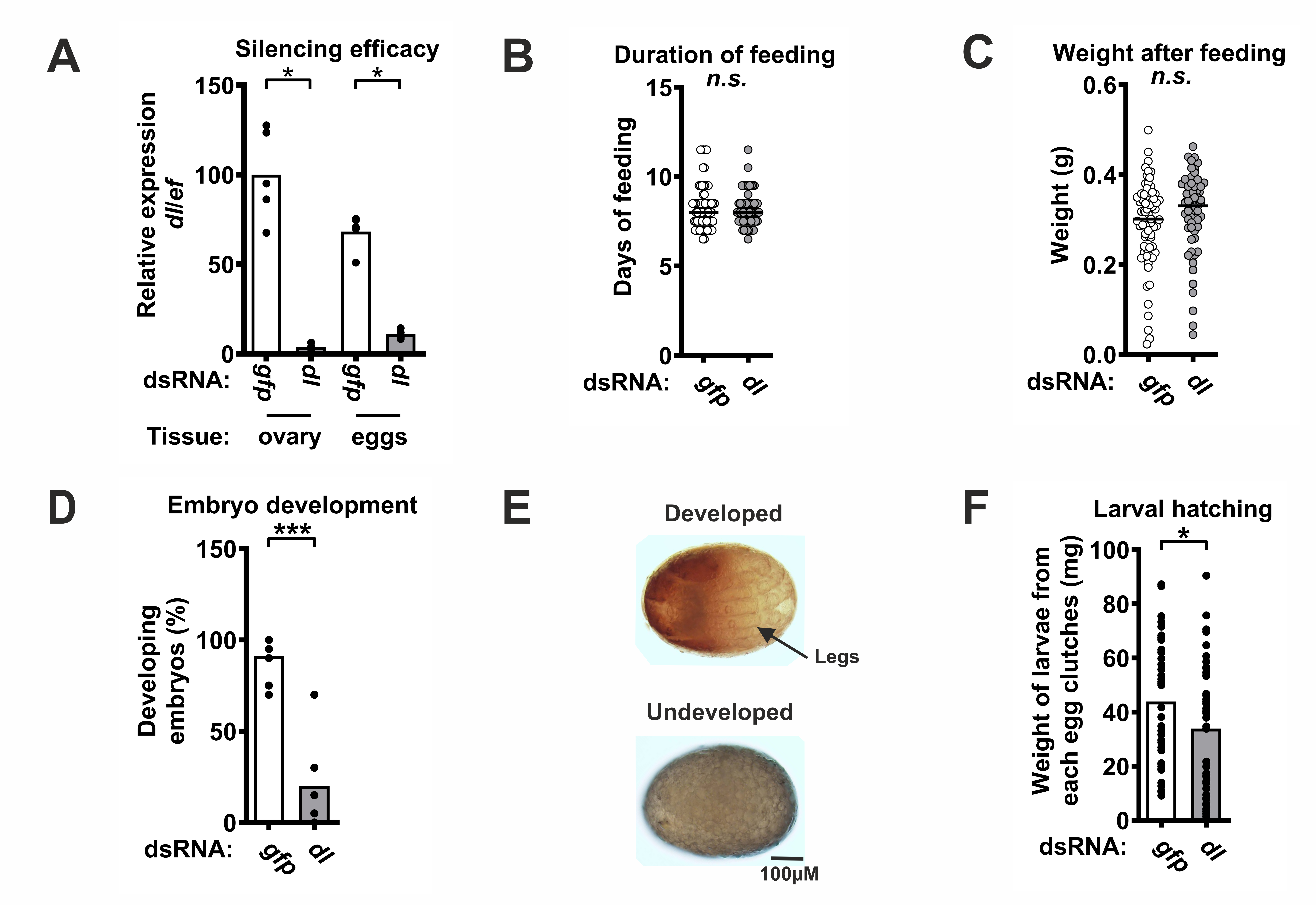

Supplement: S2 Fig — (A) Efficacy of dorsal silencing by RNAi in ovaries of half-fed adult females and laid eggs measured by qRT-PCR. Each dot represents a pool of five ovaries or egg clutches. (B) Duration of feeding of adult females. (C) Weight of fully-fed adult females. Results include data from three biological replicates. (D) Effect of gene silencing on development of tick embryo in eggs six weeks after egg laying. Twenty eggs from one clutch were analyzed for embryo development in each replicate. (E) A representative microscopic image of a developed and an undeveloped egg. (F) Total weight of all larvae hatched from the individual egg clutches. Each dot represents one clutch. dl = dorsal. *P ≤ 0.05; ***P ≤ 0.001; n.s. = not significant P ≥ 0.05. (TIF) [file ppat.1012743.s002.tif]

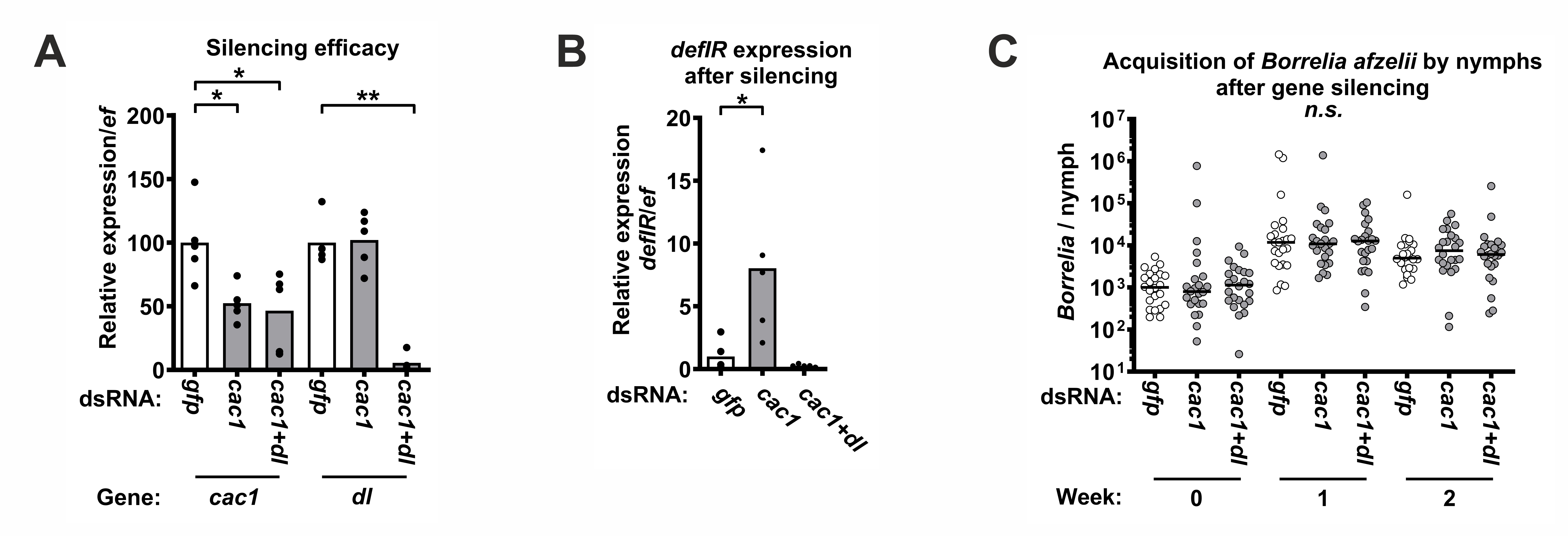

Supplement: S4 Fig — (A) Efficacy of gene silencing in the fully-fed nymphs (whole bodies) measured by qRT-PCR. Each dot represents a single nymph. (B) Relative expression (qRT-PCR) of defIR in the dsRNA-injected, fully-fed nymphs. Each dot represents a pool of five nymphs. Gene expression in the dsGFP control was set as 1. (C) Absolute number (qRT-PCR) of B. afzelii in the fully-fed nymphs pre-injected with dsRNA analyzed 0, 1, and 2 weeks after detachment. cac1 = cactus1, dl = dorsal, defIR = defensin. *P ≤ 0.05; **P ≤ 0.01; n.s. = not significant P ≥ 0.05. (TIF) [file ppat.1012743.s004.tif]

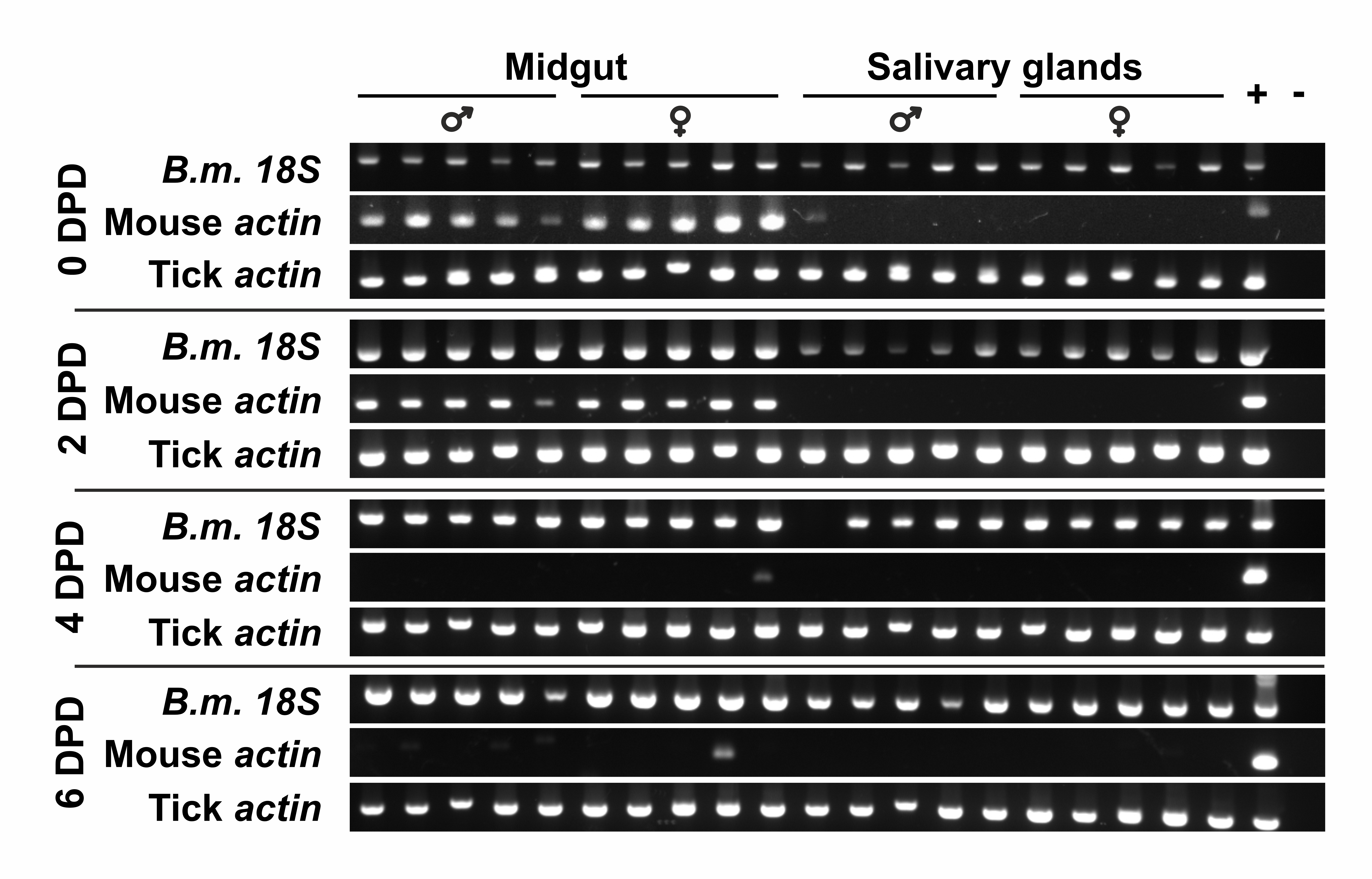

Supplement: S5 Fig — Each sample represents a single tick. (TIF) [file ppat.1012743.s005.tif]

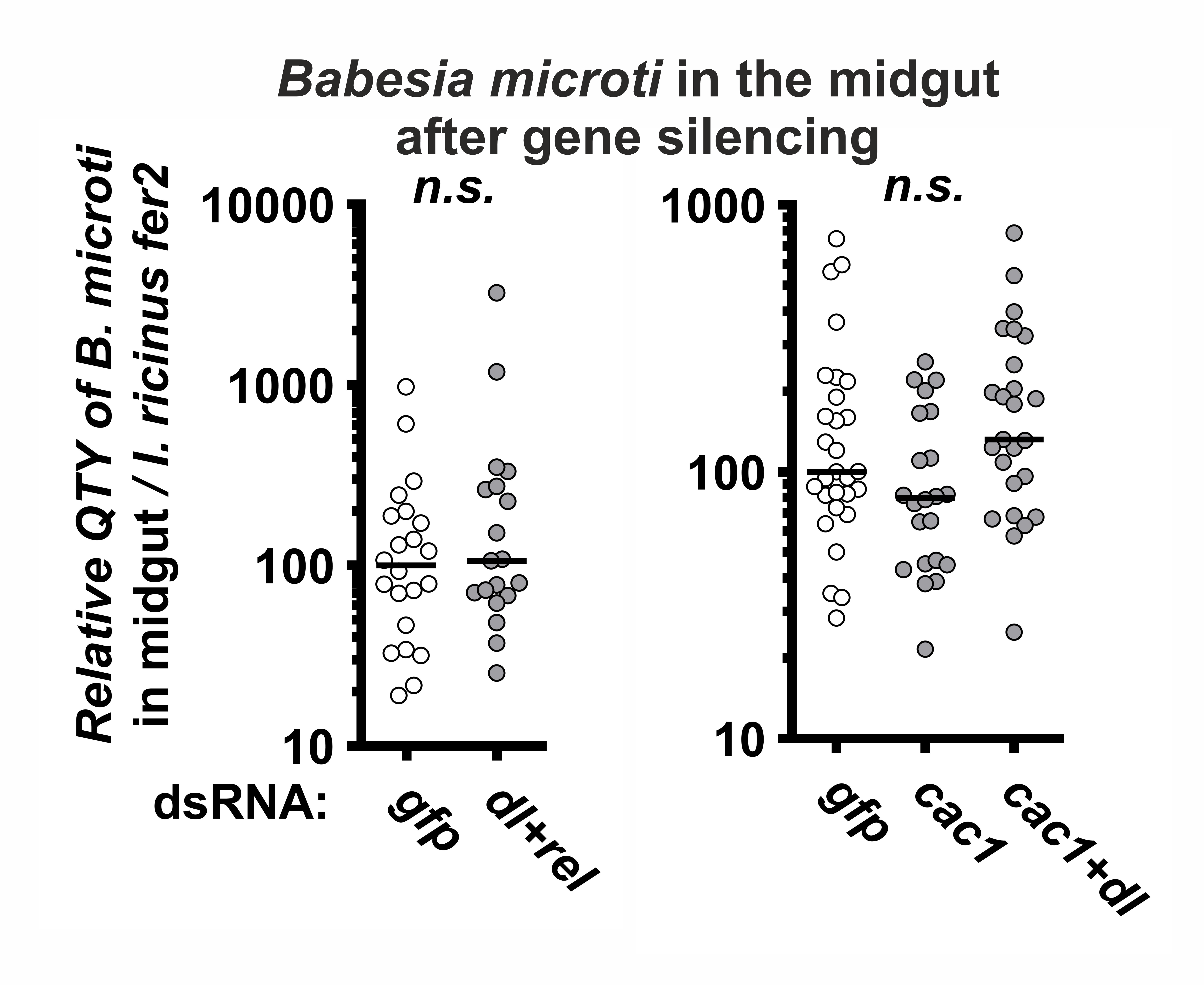

Supplement: S6 Fig — Relative quantity (qRT-PCR) of B. microti in the midgut of nymphs pre-injected with dsRNA analyzed 6 days post detachment (DPD). The results were normalized to I. ricinus ferritin 2 (fer2). dl = dorsal, rel = relish, cac1 = cactus1. n.s. = not significant P ≥ 0.05. (TIF) [file ppat.1012743.s006.tif]

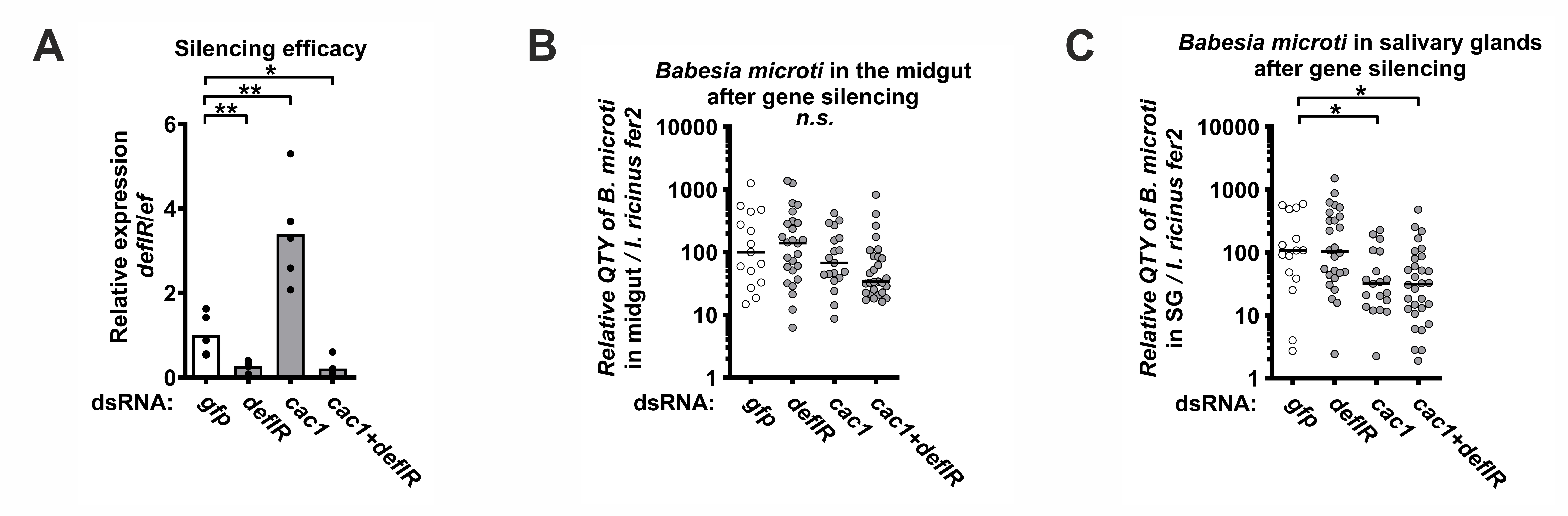

Supplement: S7 Fig — (A) Efficacy of defIR silencing in the fully-fed nymphs (whole bodies) measured by qRT-PCR. Each dot represents a pool of five nymphs. (B-C) Relative quantity (qRT-PCR) of B. microti in the midgut (B) and salivary glands (C) of nymphs pre-injected with dsRNA analyzed 6 DPD. The results were normalized to I. ricinus ferritin 2 (fer2). defIR = defensin, cac1 = cactus1. *P ≤ 0.05; **P ≤ 0.01; n.s. = not significant P ≥ 0.05. (TIF) [file ppat.1012743.s007.tif]

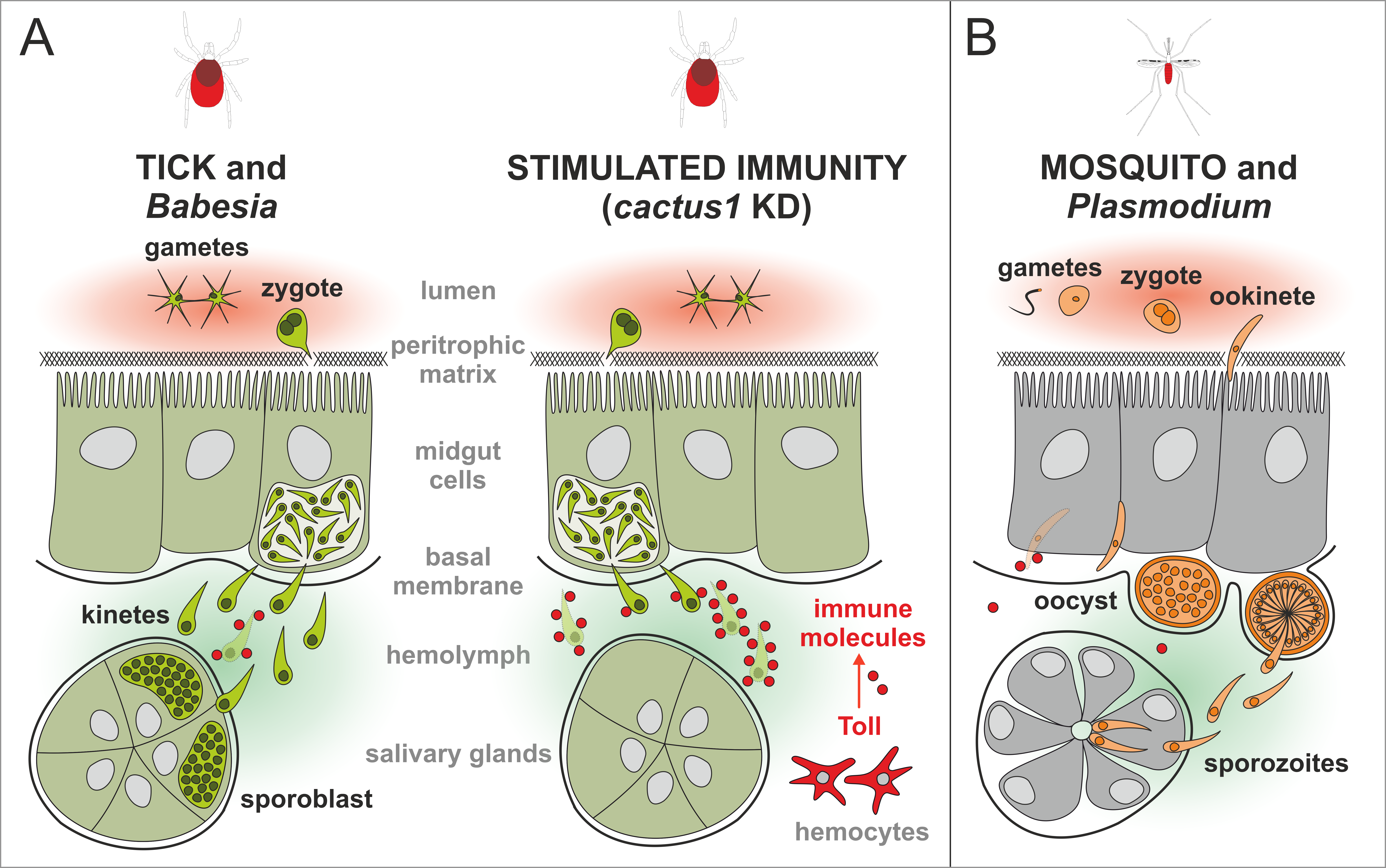

Supplement: S8 Fig — (A) Development of B. microti in I. ricinus. The tick acquires the parasites by feeding (which takes several days) on host blood infected with B. microti. During the sexual stage of the parasite in the midgut lumen of the tick, the Babesia gametes release from the infected red blood cells and fuse to form motile zygotes (ookinetes). The zygotes mechanically penetrate the newly-forming peritrophic matrix and enter the midgut cells to multiply and produce kinetes. The kinetes leave the midgut cells and infect the salivary glands via the hemolymph. In the hemolymph, the kinetes are exposed to the damaging effect of immune molecules (red dots), which are mainly secreted by hemocytes. In the salivary glands, the kinetes transform into sporoblasts, which survive the molting of the tick and remain until the next blood meal, where they mature into sporozoites that invade the host red blood cells. Through the Toll pathway, the tick immune system can balance the number of Babesia parasites (which was shown in our experiments by silencing cac1 and cac1+dorsal) and thus determine the tick vector capacity. (B) Development of Plasmodium in the mosquito. Ookinetes, formed by the fusion of gametes, cleave chitin in the peritrophic matrix with the help of chitinase and then traverse the midgut cells to form oocysts between the cells and basal membrane. Here the ookinetes are exposed to attack by the mosquito immune molecules (e.g., thioester protein 1 (TEP1)). After a few days, the sporozoites break through the oocyst wall, are released into the hemolymph and migrate to the salivary gland. They accumulate in the secretory cavities and salivary gland ducts until the next feeding. (TIF) [file ppat.1012743.s008.tif]
