# Supplementary material for: Activation of the tick Toll pathway to control infection of Ixodes ricinus by the apicomplexan parasite Babesia microti
Source: PLoS Pathog. 2024 Dec 16;20(12):e1012743. doi: 10.1371/journal.ppat.1012743 (PMC11649134; doi:10.1371/journal.ppat.1012743)
Supplement: S1 Table — Restriction sites for ApaI/XbaI are underlined. (PDF) [file ppat.1012743.s009.pdf]

Method	Target	NCBI reference	ID	Sequence (5´→3´)
RNAi	*Ixodes ricinus*: *dorsal*	GIXL01005860	IR-11	ATGGGCCCCAAGGTGATGCACCACCAGG
			IR-12	ATTCTAGACAGATGGCCACCTGCTTGTG
	I. ricinus: *relish*	GIXL01012869	IR-15	ATGGGCCCCCTCGCCATGCCCATCTCC
			IR-16	ATTCTAGAGAACTGGATGCGCACCAGG
	I. ricinus: *cactus1*	GIXL01010210	IR1402	ATTCTAGAGACAGACGGCCCTACACGTG
			IR1403	ATGGGCCCCTGGAGCCGGAGCGTACCG
	I. ricinus: *cactus2*	GIXL01004049	IR-33	ATGGGCCCAACCCTCGAGCTCCAGTACG
			IR-34	ATTCTAGAGCGATGGCGTGGTAGAGCG
	I. ricinus: *defIR*	GANP01012097	IR1404	ATGGGCCCGGTCCTTGCTGTCTCGCTTGC
			IR1405	ATTCTAGACTTCACACAGATGCAGGTCC
	I. ricinus: *myd88*	GIXL01005445	IR-256	ATGGGCCCCTCAGCAGAAACTGATTCC
			IR-257	ATTCTAGACTGGCGTTGCCATACTCTG
qRT-PCR (cDNA)	*I. ricinus*: *dorsal*	GIXL01005860	IR13	GACGTGCACCTCCTCTTGA
			IR14	CTCCGGATCCCTCTCGTT
	I. ricinus: *relish*	GIXL01012869	IR1408	ACCCTCTGCTGCGTCTACTC
			IR1409	TCCTCGTCCTCCTCAAAGAA
	I. ricinus: *cactus1*	GIXL01010210	IR1400	AGATCTATGCGCAGGACGA
			IR1401	GTTCCTCAGATCCAGCAGGT
	I. ricinus: *cactus2*	GIXL01004049	IR35	GTGGCCCGCATAGTCTGT
			IR36	CAGAGAGCAGCCTCGACAC
	I. ricinus: *defIR*	GANP01012097	IR1331	GGAGAAAAACAGCTCGTTCG
			IR1332	TTCAGAAAATTGCCGCAGTA
	I. ricinus: *elongation factor*	GU074769	IR524	ACGAGGCTCTGACGGAAG
			IR525	CACGACGCAACTCCTTCAC
	I. ricinus: *tep1*	MT779791	IR135	GAAAGCGCACCCTCAAGAT
			IR136	AAGTCTGATGGAAGCTCGATG
	I. ricinus: *c3-1*	MT779792	IR131	AGGTGAACGAAACAGCACCT
			IR132	CTTCCAGAAGGGCCACAA
	I. ricinus: *c3-2*	MT779793	IR952	TGCAGCTGTTCAGGTGAAAT
			IR953	ATGTCCAAGGCAAGTGTTCC
	I. ricinus: *c3-3*	MT779794	IR137	GGGAGTCCCGTGTCAGTAGA
			IR138	AGGTAGTCGAGCACAAAGACG
	I. ricinus: *α2m1*	MT779788	IR131	AGGTGAACGAAACAGCACCT
			IR132	CTTCCAGAAGGGCCACAA
	I. ricinus: *α2m2*	MT779789	IR952	TGCAGCTGTTCAGGTGAAAT
			IR953	ATGTCCAAGGCAAGTGTTCC
	I. ricinus: *α2m3*	MT779790	IR137	GGGAGTCCCGTGTCAGTAGA
			IR138	AGGTAGTCGAGCACAAAGACG
	I. ricinus: *mcr1*	MT779795	IR954	TTCAAAGGGGACCGTGTTAC
			IR955	GGAGCCATCTCTGGTGAAAG
	I. ricinus: *mcr2*	MT779796	IR147	CAACGTGGAGACCACATCCT
			IR148	TGTTGAGCCACTCGACAATC
	I. ricinus: *microplusin*	GBIH01001600	IR1337	GCTCTCTCGCACTCTTCGTT
			IR1338	GCGTCATCAAGATGGCAGTA
	I. ricinus: *ixoA*	AY341424	IR3	AGGGAACATCGGTCTATTGC
			IR4	TGGCCTCTGTTCTGAATAACG
	Babesia microti: *ama-1*	JX488467	IR443	ATTCAACTGCGCCTCCTATG
			IR444	TGGATTAGTTGCAACGGAGA
	I. ricinus: *myd88*	GIXL01005445	IR258	GCTGGCGTGCTAAAGTACAA
			IR259	CTGGGGAAAATATGATGATGG
qRT-PCR (DNA)	*I. ricinus*: *fer2*	EU885951	IR92	TCAGCTCATGGACTTCATCG
			IR93	ATTCGCTGCAGCTTGTCAAT
	Borrelia spp. *flagellin*	(1)	IR1345	AGCAAATTTAGGTGCTTTCCAA
			IR1346	GCAATCATTGCCATTGCAGA
			Fla Probe1	TGCTACAACCTCATCTGTCATTGTAGCATCTTTTATTTG
	Mus musculus: *actin*	(2)	MM-ACT-F	AGAGGGAAATCGTGCGTGAC
			MM-ACT-R	CAATAGTGATGACCTGGCCGT

			MM-ACT-PROBE	CACTGCCGCATCCTCTTCCTCCC
PCR	*Borrelia* spp. *flagellin*	(1)	IR1345	AGCAAATTTAGGTGCTTTCCAA
			IR1346	GCAATCATTGCCATTGCAGA
	I. ricinus: *actin*	AJ889837	IR1558	CTACGAAGGGTACGCTCTGC
			IR1559	GACTCGTCGTATTCCTGCTTG
	M. musculus: *actin*	(2)	MM-ACT-F	AGAGGGAAATCGTGCGTGAC
			MM-ACT-R	CAATAGTGATGACCTGGCCGT
Nested PCR	*Babesia* spp.: *18S*	(3)	IR270-F1	AACCTGGTTGATCCTGCCAGTAGTCAT
			IR275-R1	GAATGATCCTTCCGCAGGTTCACCTAC
			IR272-F2	GYYTTGTAATTGGAATGATGG
			IR273-R2	CCAAAGACTTTGATTTCTCTC

1. Schwaiger M, Peter O, Cassinotti P (2001) Routine diagnosis of *Borrelia burgdorferi* (sensu lato) infections using a real-time PCR assay. *Clin Microbiol Infect* 7(9):461–469.
2. Dai J, et al. (2009) Antibodies against a Tick Protein, Salp15, Protect Mice from the Lyme Disease Agent. *Cell Host Microbe* 6(5):482–492.
3. Malandrin L, Jouglin M, Sun Y, Brisseau N, Chauvin A (2010) Redescription of *Babesia capreoli* (Enigk and Friedhoff, 1962) from roe deer (*Capreolus capreolus*): Isolation, cultivation, host specificity, molecular characterisation and differentiation from *Babesia divergens*. *Int J Parasitol*. doi:10.1016/j.ijpara.2009.08.008.
