# Supplementary material for: Activation of the tick Toll pathway to control infection of Ixodes ricinus by the apicomplexan parasite Babesia microti
Source: PLoS Pathog. 2024 Dec 16;20(12):e1012743. doi: 10.1371/journal.ppat.1012743 (PMC11649134; doi:10.1371/journal.ppat.1012743)
Supplement: S1 Data — (PDF) [file ppat.1012743.s010.pdf]

Target	Bait (*Drosophila melanogaster*)	NCBI Accesion number Bioproject PRJNA657487	E-value	Name	Note	Number of transcripts	Number of genes
**Toll pathway**							
**PGRP-SA**	NP_572727.1	GIXL01004145	7.00E-33			25	**25**
		GIXL01000128	3.00E-30				
		GIXL01017829	3.00E-27				
		GIXL01016007	6.00E-27				
		GIXL01002576	6.00E-27				
		GIXL01026083	3.00E-26				
		GIXL01010148	3.00E-26				
		GIXL01002130	4.00E-26				
		GIXL01002573	1.00E-25				
		GIXL01002567	1.00E-25				
		GIXL01018184	2.00E-25				
		GIXL01015138	3.00E-25				
		GIXL01005998	7.00E-25				
		GIXL01028973	2.00E-24				
		GIXL01028256	2.00E-24				
		GIXL01002136	2.00E-24				
		GIXL01014311	2.00E-23				
		GIXL01000483	7.00E-23				
		GIXL01026060	5.00E-21				
		GIXL01006731	2.00E-19				
		GIXL01009187	3.00E-17				
		GIXL01015145	4.00E-16				
		GIXL01014366	3.00E-13				
		GIXL01008185	1.00E-08				
		GIXL01011993	2.00E-08				
**GNBP1**	NP_524142.2	absent				0	**0**
**GNBP3**	NP_523986.2	absent				0	**0**
**Spz**	NP_524526.1	GIXL01009369	4.00E-15			5	**4**
		GIXL01013401	8.00E-12		Isoforms		
		GIXL01028106	1.00E-10		Isoforms		
		GIXL01003183	2.00E-10				
		GIXL01008410	1.00E-09				
**Toll**	NP_524518.1	GIXL01017769	e-120			7	**7**
		GIXL01018393	1.00E-75				
		GIXL01023824	6.00E-74				
		GIXL01007216	8.00E-72				
		GIXL01008665	4.00E-64				
		GIXL01009165	7.00E-25				
		GIXL01019359	2.00E-18				
**MyD88**	NP_610479.1	GIXL01005445	4.00E-17			1	**1**
**Tube**	XP_008198084 *	GIXL01013735	2.00E-46			1	**1**
**Pelle**	NP_476971.1	GIXL01026675	6.00E-67			1	**1**
**Pellino**	NP_524466	GIXL01012339	e-167			1	**1**
**Dorsal**	NP_724052.1	GIXL01005860	3.00E-97	Dorsal	Isoforms	3	**1**
		GIXL01018622	6.00E-96		Isoforms		
		GIXL01019808	8.00E-96		Isoforms		
**Dif**	NP_523589.2	absent				0	**0**
**Cactus**	NP_723960.1	GIXL01010210	2.00E-29	Cactus1		2	**2**
		GIXL01004049	1.00E-18	Cactus2			
**Cactin**	NP_523422.4	GIXL01024193	0			1	**1**
**Imd pathway**							
**PGRP-LB**	NP_650079.1	absent				0	**0**
**PGRP-SC**	NP_610410.1	absent				0	**0**
**PGRP-LC**	NP_729468.2	absent				0	**0**
**PGRP-LE**	NP_573078.1	absent				0	**0**
**Diap2**	NP_477127.1	absent				0	**0**
**Bendless**	NP_511150.1	GXP_Contig_20498_2	2.00E-74			8	**8**
		GXP_Contig_30742	8.00E-29				
		GXP_Contig_20302_3	3.00E-25				
		GXP_Contig_31304_1	3.00E-23				
		GXP_Contig_8730_1	3.00E-23				
		GXP_Contig_5591_1	2.00E-21				
		GXP_Contig_27381_3	1.00E-20				
		GXP_Contig_22038_7	1.00E-08				
**Uev1a**	NP_647959.1	GXP_Contig_31594_1	6.00E-59			2	**2**
		GXP_Contig_32425_1	4.00E-48				
**Effete**	NP_731941.1	GXP_Contig_24685_1	1.00E-85			21	**21**
		GXP_Contig_32618	6.00E-83				
		GXP_Contig_33137_1	3.00E-52				
		GXP_Contig_30742_12	3.00E-52				
		GXP_Contig_30741_1	3.00E-52				
		GXP_Contig_33368_1	2.00E-31				
		GXP_Contig_23538_4	2.00E-28				
		GXP_Contig_31607_2	6.00E-27				
		GXP_Contig_20302_3	5.00E-25				
		GXP_Contig_2304_1	6.00E-22				
		GXP_Contig_30735_2	4.00E-20				
		GXP_Contig_31996_1	5.00E-20				
		GXP_Contig_31205_1	1.00E-16				
		GXP_Contig_31204_6	1.00E-16				
		GXP_Contig_26978_4	2.00E-15				
		GXP_Contig_29396_1	4.00E-15				
		GXP_Contig_33061_1	8.00E-11				
		GXP_Contig_18215_2	4.00E-10				
		GXP_Contig_31594_1	1.00E-07				
		GXP_Contig_5833_1	7.00E-06				
		GXP_Contig_7795_2	2.00E-05				
**IMD**	NP_573394.1	absent				0	**0**
**TAK1**	NP_524080.1	GIXL01012856	8.00E-80			1	**1**
**TAB1**	NP_611408.2	absent				0	**0**

**IKKgamma**	NP_523856.2	GIXL01008857	3.00E-09			1	**1**
**IKKbeta**	NP_524751.3	GIXL01002279	1.00E-42			1	**1**
**Fadd**	NP_651006.1	absent				0	**0**
**Dredd**	NP_477251.3	absent				0	**0**
**Relish**	NP_477094.1 **	GIXL01012869	5.00E-49	Relish		1	**1**
**Caspar**	NP_611080.1	GIXL01011135	2.00E-91			1	**1**

* = *Tribolium castaneum*

** = ANK domains missing
