# Supplementary material for: Activation of the tick Toll pathway to control infection of Ixodes ricinus by the apicomplexan parasite Babesia microti
Source: PLoS Pathog. 2024 Dec 16;20(12):e1012743. doi: 10.1371/journal.ppat.1012743 (PMC11649134; doi:10.1371/journal.ppat.1012743)
Supplement: S1 Text — (PDF) [file ppat.1012743.s012.pdf]

AAEL007696_Rel1A_*Aedes aegypti*

AAEL006930_Rel1B_*Aedes aegypti*

XP_310177_Rel1_*Anopheles gambiae*

NP_724052_Dorsal_*Drosophila melanogaster*

GEFM01002316_Dorsal_*Ixodes ricinus*

JAA63771_Rel1_*Rhipicephalus pulchellus*

XP_025016999_Rel1_*Tetranychus urticae*

AAZ40333_Rel1_*Carcinoscorpinus rotundicauda*

NP_523589_Dif_*Drosophila melanogaster*

AAEL007624_Rel2_*Aedes aegypti*

XP_308995_Relish_*Anopheles gambiae*

NP_477094_Relish_*Drosophila melanogaster*

ABC75034_Relish_*Carcinoscorpinus rotundicauda*

XP_025016550_Relish_*Tetranychus urticae*

GIDG01030620_Relish_*Ixodes ricinus*

JAA61285_Relish_*Rhipicephalus pulchellus*

AJA91066_Relish_*Dermacentor variabilis*
