# Supplementary material for: Activation of the tick Toll pathway to control infection of Ixodes ricinus by the apicomplexan parasite Babesia microti
Source: PLoS Pathog. 2024 Dec 16;20(12):e1012743. doi: 10.1371/journal.ppat.1012743 (PMC11649134; doi:10.1371/journal.ppat.1012743)
Supplement: Attachment — Submitted filename: Rebuttals PPATHOGENS-D-24-01540 Hajdusek.docx [file ppat.1012743.s013.docx]

**Point-by-point response to reviewer’s questions**

**Part II. Major Issues: Key Experiments Required for Acceptance**

**Reviewer #1**

Major Issue 1: Cact1 is expressed in ovaries and hemocytes; - makes sense that it is involved in development. Cac2 is expressed predominantly in hemocytes (line 133). Yet it is only Cact1 that is targeted in all their studies. I could not find an explanation for why only Cact1 was being targeted. What would be the phenotype in the context of Cact2 targeting? In line 168, they emphasize the cact1-dorsal module in the Toll pathway but given that cact2 is expressed in hemocytes -showing no data on cact2 leaves a major gap in the conclusions presented. This would shed further insights into this pathway.

Our response to Major Issue 1: We show that the core genes of the tick Toll pathway, *cactus1* and *dorsal*, are expressed primarily in ovaries (suggesting a role in development) and hemocytes (suggesting a function in immunity) (Fig 1D). Unlike the Toll pathway transcripts, *cactus2* is expressed exclusively in hemocytes. Importantly, the amino acid sequence of Cactus2 lacks the PEST sequence (P/E/S/T-rich sequence, a signal peptide for protein degradation) found in the Cactus proteins of other animals and in Cactus1 of *Ixodes ricinus*. The PEST sequence is essential for phosphorylation, dissociation from NF-κB (nuclear factor kappa-light-chain-enhancer of activated B cells; Dorsal) (Fig 1B), and subsequent degradation of I-κB (inhibitor of κB; Cactus) proteins after activation of the Toll pathway (Fig 1A). In addition, Cactus2 contains an unusual nuclear localization signal that predicts its presence in the nucleus (Lines 122-125). While Cactus2 possesses ankyrin repeats typical of other Cactus proteins, the absence of the PEST sequence and the presence of the nuclear signal likely prevent it from functioning in the cytoplasm as a typical I-κB inhibitor that binds Dorsal. Because of these atypical features, we excluded Cactus2 from further analysis and focused exclusively on Cactus1. We have revised the sentence in the manuscript (now Lines 192-199) to clarify this decision. The function of Cactus2 remains unknown, and we are conducting studies to clarify its role in tick physiology.

Major Issue 2: Also, only dorsal is targeted in the development phenotype experiments. Would be important to know the impact of Cactus 1 targeting in this, given that this is also predominant in the ovaries. In the development part of the manuscript, Fig 2 notes that embryo development was dramatically reduced in Fig 2B (Fig 2C -not clear what was being observed as legs? a better image is requested), however this is not consistent with the larval hatching impact in 2D. Was egg laying delayed in anyway? Was egg development monitored over time? This leg development parameter as a score of development could be monitored over time to score for the developmental delay. Without this information, the inconsistency in the strength of the impact between Fig 2B and 2D is confusing.

Our response to Major Issue 2: We suggested, that silencing of *cactus1* by RNAi would likely result in a developmental phenotype similar to that observed in the silencing of *dorsal*, and therefore may not provide additional new insights (DOI: 10.1371/journal.pbio.1000434). Furthermore, our results showed that *dorsal* was more effectively targeted by RNAi in ticks than *cactus1* (Fig 2E), possibly due to feedback loops in *cactus1* transcription (DOI: 10.3389/fimmu.2019.01785). Therefore, based on studies in other arthropods, we focused on knockdown and phenotype analysis of *dorsal*. The feeding experiment with *cactus1* silencing in adult ticks, including egg laying and larval hatching, requires at least three months, which exceeds the time frame of the rebuttals.

In Fig 2C, we have indicated with an arrow that the developing embryos in the control group already had visible legs (clearer when magnified), a typical feature of embryonic development in ticks (DOI: 10.1002/dvg.22717). Given the inconsistencies in the phenotypes observed in Fig 2B and D, we agree that it remains unclear whether development was trully blocked or merely delayed. Statistically, however, *dorsal* knockdown resulted in a lower percentage of developing embryos (six weeks after oviposition) and consequently a lower number of hatched larvae. The period of six weeks after oviposition was accurately identified as optimal for monitoring egg development, as it lies between the appearance of microscopic signs of embryonic development and egg hatching. The larvae hatched without visible morphological anomalies.

Our original intention was to present the role of the Toll pathway in its entirety. While the data on tick development contribute to the overall understanding of the manuscript, we agree that they are not central and could distract from the role of the Toll pathway in the tick immune system. Therefore, we decided to move Fig 2A-D to the Supplementary materials (now Fig S2) and added the data on the length of feeding and weight of adult females after *dorsal* silencing. We hope that these changes will provide a more complete picture of the importance of the Toll pathway in tick development and better clarify the focus of the manuscript. The references to this Supplementary figure and its legend have been changed accordingly.

Major Issue 3: In injection experiments with E. coli or M. luteus, the authors knockdown the expression of myd88 and wait for 2 weeks before injecting with the pathogens. Unclear why the wait was required. Also not explained in the Methods section.

Our response to Major Issue 3: After injecting the dsRNA, we usually let the nymphs rest in a humid chamber for three days (Lines 442-443). However, in this particular case, we found that it was better to let the nymphs rest for two weeks. This change was in response to the observed stimulation of the tick immune system due to the needle injury one day after injection (Fig 3C: *gfp* in PBS-injected group). After two weeks, the expression of immune genes (*defIR*) had almost returned to baseline levels (Fig 3C: *gfp* in Untreated group), suggesting that such treated ticks were better prepared for subsequent immune challenges. Importantly, the expression of genes that were silenced by RNAi remained effectively suppressed during this extended period.

Major Issue 4: In the E. coli, M. luteus experiments -did they check for any readouts in the IMD pathway? AMP such as Ctenidin was shown to be activated by the IMD pathway (ONeal et al, 2023).

Our response to Major Issue 4: Our study focused primarily on the tick Toll pathway rather than the IMD pathway, which has already been studied in detail in several valuable publications (DOI: 10.1073/pnas.2208673120; DOI: 10.1038/ncomms14401; DOI: 10.1073/pnas.1808905116; DOI: 10.1128/mbio.00703-22). As mentioned in our manuscript, the IMD pathway is not fully conserved in ticks and lacks several key components (Fig 1A and S1). Alternative mechanisms for the activation of this pathway have been proposed by other groups (DOI: 10.1038/ncomms14401; DOI: 10.1073/pnas.2208673120). In particular, we show that tick Relish lacks the essential ankyrin domains required for its activation and that the protease responsible for cleavage of Relish (DREDD) has not yet been identified in the tick genome or transcriptome. These aspects of the IMD pathway certainly require further investigation.

Regarding the *ctenidin* gene, O'Neal et al., 2023 (DOI: 10.1073/pnas.2208673120) showed that its expression is downregulated by silencing *relish* and *croquemort* (a newly proposed transmembrane activator of the IMD pathway), as noted by Reviewer 1. The authors also showed that silencing of *dorsal* had no effect on the expression of *ctenidin*. However, it is important to emphasize that the regulation of *ctenidin* by components of the IMD pathway has not been genetically demonstrated, in contrast to *defIR* in the Toll pathway, as shown in our publication. For these reasons, and because our study focused on the Toll signaling pathway, we did not include *ctenidine* in our expression measurements following bacterial injection or activation of the Toll signaling pathway.

However, in response to the reviewer’s comments, we analyzed the expression of *ctenidin-1* using our cDNA sets. The homologous nucleotide sequence to *Ixodes scapularis* *ctenidin- 1* (XM_029986701) was retrieved from the *I. ricinus* genome database (Iricinus_Scaffold_3 Iricinus_Scaffold_3:173346216..173346477) on the BIPAA server (https://bipaa.genouest.org/sp/ixodes_ricinus/; https://www.biorxiv.org/content/10.1101/2024.02.29.581698v1.full). Since the nucleotide sequence in the primer region showed 100% homology, we used the same primers published in O'Neal et al. 2023. The same set of cDNAs as in Fig 3A and 2F was used for qRT-PCR analysis. To confirm the identity of the amplicon, the PCR product was cloned into a pGEM-T Easy Vector (Promega) and sequenced (100% identity). Our results showed that *ctenidin-1* was upregulated in *Ixodes ricinus* by injection of *Escherichia coli* (Gram-negative bacteria), but not by knockdown of *cactus1* (Reviewer Fig 1). This suggests that although *ctenidin-1* is a putative immune gene, its expression is not regulated by the Toll pathway. We have decided not to include these data on *ctenidin-1* expression in the manuscript.

**Reviewer Figure 1.** Expression of *ctenidin-1* is induced by injection of microbes but not by activation of the Toll pathway. (A) Relative expression (qRT-PCR) of *ctenidin-1* in the unfed nymphs 24 hours after injection of microbes. Each dot represents a pool of ten nymphs. (B) Relative expression of *ctenidin-1* in the dsRNA-injected, fully fed nymphs. Each dot represents a pool of five nymphs. Gene expression in the PBS (A) and dsGFP (B) control was set as 1. *E. coli* = *Escherichia coli*, *M. luteus* = *Micrococcus luteus*, *C. albicans* = *Candida albicans*, *cac1* = *cactus1*, *dl* = *dorsal*, *rel* = *relish*. **P ≤ 0.01; *n.s.* = not significant P ≥ 0.05.

Major Issue 5: Upon knockdown of Myd88 and injection of E. coli, the authors do not monitor tick survival, and this further begs the question as to whether Toll pathway restricts such bacteria in nature. This also raises another aspect of this study – using bacteria such as E. coli and M. luteus may not really be physiologically relevant.

Our response to Major Issue 5: *Escherichia coli* (Gram-negative bacteria) and *Micrococcus luteus* (Gram-positive bacteria) are routinely used to characterize immunity in the *Drosophila* model, where they specifically activate the IMD and Toll pathways, respectively (DOI: 10.1038/nri2194). To place tick immunity in an evolutionary context with other arthropods, we wanted to use the same model bacterial strains for our experiments. Injection of these bacteria, either alone or after silencing of *myd88*, had no lethal effect on the ticks 24 hours after injection. Furthermore, these bacteria only acted as stimulants of specific signaling pathways that present their pathogen-associated molecular patterns (PAMPs) to the tick immune system. We are aware of the limitations of using these model microbes and realize that the biology of tick-specific pathogens (DOI: 10.1016/j.jip.2006.05.006; DOI: 10.1016/j.dci.2021.104234) may differ considerably.

Major Issue 6: Further, the authors seem to make abrupt jumps from one component of the pathway to the other. Why did they choose to knockdown myd88 in the E. coli/M. luteus experiments, but switched to dorsal/cactus in the Borrelia /Babesia experiments?

Our response to Major Issue 6: We have not chosen the components arbitrarily, but have carefully selected those that best answer our specific scientific questions. Knockdown of *myd88* is an established approach in the study of Toll signaling in arthropods and their immune responses to microbes (DOI: 10.1038/ni747). After characterizing the regulatory cassette Dorsal-Cactus1 in *Ixodes ricinus*, we aimed to demonstrate involvement of these proteins in the Toll pathway. As mentioned in the manuscript (Lines 183-184), MyD88 binds specifically to Toll receptors and is therefore unique to the Toll pathway. It is important to note that the Dorsal transcription factor may not be exclusively involved in Toll signaling and could also be activated by other pathways. By selectively silencing *myd88* in ticks that were subsequently infected with different microbes, we were able to confirm that activation of the Dorsal-Cactus1 cassette is indeed regulated by the Toll signaling pathway. This is a crucial finding that forms the basis for future elucidation of the entire tick Toll pathway by targeting individual components in microbe-infected ticks and evaluating the responses via the readout gene *defIR*. We believe that this point is discussed comprehensively in the manuscript.

In contrast, in our experiments with *Borrelia* and *Babesia*, we silenced *cactus1* or *cactus1*+*dorsal* to either upregulate or completely block the Toll pathway. Silencing of *myd88* in this context would only mimic knockdown of *dorsal*, but would not activate the Toll pathway and would likely lead to similar results as silencing of *dorsal*+*relish* in Fig 3F and 4F.

Major Issue 7: In the Babesia experiments, it is unclear what the purpose of the IFA and EM pictures are for; perhaps these images are merely to emphasize the model? Babesia experiments just leave the conclusion to much “speculation” on the part of the reader. While Babesia infection does not alter the DeflR transcript levels (just as in the case of Borrelia), knockdown of cactus provides decreased burden of Babesia in the salivary glands. Since the cacust1-dorsal module did not impact any of the TEP-like components in tick hemocytes, are the authors suggesting that Babesia parasites are being killed by DeflR? This is very speculative. If defensin like AMPs can be shown to kill Babesia in vitro-then the observations can be more mechanistically interpretable, but no such plausible mechanism is provided.

Our response to Major Issue 7: The purpose of the IFA and EM study of salivary glands from nymphs fed on *Babesia*-infected mice (Fig 4B and C) was to confirm the previous molecular detection of *Babesia* in this tissue (Fig 4A) and thus verify the successful acquisition of *Babesia* by the nymphs in our newly established laboratory model. Subsequently, the effect of Toll pathway modulation on *Babesia* infection could be assessed by quantification of *Babesia* DNA in the salivary glands alone. The red arrows in Fig 4B and C indicate infected cells with abnormal morphology (hypertrophy). The purpose of this approach and the morphology of the infected cells are explained in the Results section (Lines 227-238).

In this study, *defensin* (*defIR*) is a gene whose overexpression is specifically regulated by the tick Toll pathway (read-out gene). However, based on research in *Drosophila* and mosquitoes (DOI: 10.1371/journal.ppat.1000098; DOI: 10.1371/journal.ppat.1002394), activation of the Toll pathway (e.g., by silencing of *cactus1*) could upregulate hundreds of genes interfering with *Babesia* infection in ticks. While we did not have transcriptomic data following silencing of *cactus1* for *I. ricinus*, we focused on previously described immune genes and found that among them, expression of *defIR* was specifically driven by the Toll pathway (Fig 2F). However, this does not necessarily mean that *defIR* is directly involved in the control of *Babesia* parasites during tick infection, although previous publications showed that a synthetic tick defensin similar to *defIR* (DMT3) exhibited pathogen-inhibiting activity against *Plasmodium falciparum* (DOI: 10.1016/j.dci.2015.08.001; DOI: 10.3389/fmicb.2016.01682). Consistent with this, upregulation of Defensin A by the IMD pathway reduced the development and transmission of *Plasmodium gallinaceum* in the mosquito *Aedes aegypt*i (DOI: 10.1073/pnas.1003056107).

To confirm the anti-parasitic role of *defIR* in ticks, we quantified *Babesia* infection rates in the midgut and salivary glands of *defIR*-silenced ticks and found no significant difference compared to the dsGFP control (Reviewer Fig 2). Since the expression of *defIR* is not induced by *Babesia* infection, we silenced *defIR* together with *cactus1*, whose depletion upregulates the expression of *defIR* via the Toll pathway. However, despite this combined approach, infection rates remained similar to when *cactus1* was silenced alone (Reviewer Fig 2). This suggests that although *defIR* is activated by the Toll signaling pathway, it does not play a direct role in killing *Babesia* parasites in ticks and that immune molecules other than *defIR* are involved in this process. Their identification remains an area for future investigation. These results have been added to the Supplemental data (now Fig S7) and Results (now Lines 337-341).

**Reviewer Figure 2.** The acquisition of *Babesia microti* by tick nymphs. (A) Efficacy of *defIR* silencing in the fully-fed nymphs measured by qRT-PCR. Each dot represents a pool of five nymphs. (B-C) Relative quantity (qRT-PCR) of *B. microti* in the midgut (B) and salivary glands (C) of nymphs pre-injected with dsRNA analyzed 6 DPD. *P ≤ 0.05; **P ≤ 0.01; *n.s.* = not significant P ≥ 0.05.

Major Issue 8: Lines 384-390 – very difficult to follow the rationale. Complicated further by Suppl Fig S1 that blood feeding induced DeflR expression and was not further increased upon Cac-1 silencing. The authors suggest that this is also consistent with the observation that dorsal +relish knockdown had no impact on Babesia, but dorsal +cactus did? It makes the arguments convoluted with no additional data to validate such speculations. If Dorsal-the transcription factor, induces the transcription of effector molecules dorsal +relish should also result in increased Babesia burden ?

Our response to Major Issue 8: Thank you for this comment. We recognize that the reasoning here, the sequence of experiments and their discussion were complicated and did not fully support our conclusions. For this reason, we have decided not to include Fig S5 in our revised manuscript. We can only speculate about the reasons for the different extent of *Babesia* infection in the *dorsal*+*relish* and *cac1*+*dorsal* silenced groups, as the absence of *dorsal* in both groups should lead to similar phenotypes. We hypothesize that this could be due to differences in RNAi efficiency or protein turnover, with the complete depletion of Cac1-Dorsal complexes in the *cac1*+*dorsal* group compared to their possible residual presence in the *dorsal*+*relish* group. This is certainly an area for further research. We have revised the Discussion (now Lines 473-478) to simplify the text and help the reader understand our reasoning.

Major Issue 9: For Babesia acquisition, midguts and salivary glands were dissected only from female (based on engorgement weights) nymphs, why not the male nymphs? Also, why only SG data are shown? How about the midgut burden?

Our response to Major Issue 9: The decision to focus on female nymphs in the quantification of *Babesia* infection was primarily practical. Female nymphs are larger, which allows for more accurate dissection of their salivary glands compared to males. Although we did not quantify *Babesia* infection in male nymphs by qRT-PCR, it is reasonable to assume that infection patterns would be comparable to those of females. This assumption is supported by the data in Fig 4A and S3, which show very similar infection patterns (PCR) in the salivary glands and midgut of both male and female nymphs, from 0 to 6 days post detachment (DPD).

In the context of *Babesia* infections, we quantified the number of parasites in dsRNA-treated fed nymphs using DNA-based qRT-PCR in both the salivary glands and midgut (data for the midgut were not included in the original manuscript). These results showed that stimulation of the Toll pathway had no significant effect on the number of *Babesia* parasites in the midgut (Reviewer Fig 3). However, the number of parasites in the salivary glands was significantly reduced, suggesting that tick immunity probably exerts a killing effect in the hemolymph that *Babesia* kinetes (formed in the midgut cells) must pass through on their way to the salivary glands. In response to Reviewer 1's comment, we decided to include quantification of midgut data in the Supplemental material (now Fig S6).

**Reviewer Figure 3.** The tick Toll pathway does not regulate the infection of tick nymphs by *Babesia microti* in the midgut. Relative quantity (qRT-PCR) of *B. microti* in the midgut of nymphs pre-injected with dsRNA analyzed 6 DPD. *dl* = *dorsal*, *rel* = *relish*, *cac1* = *cactus1*, *defIR* = *defensin*. *n.s.* = not significant P ≥ 0.05.

In response to Reviewer 1's comment from the Summary "In the entire manuscript the authors focus on hemocytes as key players, presumably. In the context of Borrelia and Babesia, the midguts should also be addressed. While the authors have examined Borrelia transmission in Cactus/dorsal knockout experiments, they do not address Borrelia acquisition -as the spirochetes enter the midgut to colonize the tick. Midguts are expressing these Toll components (authors show this Fig 1) – the authors have missed out on this whole important aspect of Borrelia -tick interactions. Borrelia migrates through the hemocoel during transmission, a short time when compared to its survival in the midgut over its entire time in the tick.", we quantified the amount of environmental bacteria in the midgut of fed ticks (6 DPD) using qRT-PCR in the knockdown experiments with components of the Toll pathway. These data, which were not included in the original manuscript, showed no significant differences in bacterial loads between the knockdown groups and the dsGFP controls (Reviewer Fig 4). Based on these results, we hypothesized that the Toll pathway is unlikely to have a significant effect on the acquisition of *Borrelia* that reside in the tick midgut after feeding until the next blood meal. For this reason, we decided to perform the *Borrelia* transmission experiment (Fig 3E and F) to test whether immune molecules stimulated by the Toll pathway interfere with the migration of *Borrelia* through the hemolymph to the vertebrate host.

**Reviewer Figure 4.** The tick Toll pathway does not regulate the amount of bacteria in the midgut. Relative quantity (qRT-PCR) of bacteria in the midgut of nymphs pre-injected with dsRNA analyzed 6 DPD. *cac1* = *cactus1*, *dl* = *dorsal*, *rel* = *relish*, *defIR* = *defensin*, *n.s.* = not significant P ≥ 0.05.

However, in the time we had available for the revision, we decided to carry out the experiment on the acquisition of *Borrelia* by the tick nymphs. Six-week-old C3H mice (Anlab) were infected by injection of 1x10^7^ *B. afzelii* CB43. Infection of the mice was checked three weeks after injection in the ears by PCR amplification of the *flagellin* gene. Twenty dsRNA-injected (three days of rest after dsRNA injection) nymphs of *I. ricinus* were fed on each mouse (6 mice per group) until full engorgement. The level of gene silencing was checked by qRT-PCR in five individual, fully-fed nymphs (whole bodies) and compared with the dsGFP control group. The number of spirochetes in the fully fed nymphs was quantified by qRT-PCR (*flagellin*) immediately after feeding and one and two weeks after feeding (24 nymphs per timepoint, equal number of females and males) as described previously (DOI: 10.1128/IAI.00896-18).

The injection of dsRNA led to a significantly reduced expression of genes in fully fed ticks (Reviewer Fig 5A). In addition, silencing of *cac1* resulted in a significant increase in the expression of *defIR* (indicating stimulation of the Toll pathway), while co-silencing of *cac1*+*dorsal* maintained expression at a level comparable to that of the dsGFP control (Reviewer Fig 5B). However, we could not detect a significant difference in the number of *Borrelia* between the *cac1*-silenced group, the *cac1*+*dorsal* group, and the dsGFP control (Reviewer Fig 5C). Thus, we conclude that stimulation or deactivation of the Toll signaling pathway had no effect on the acquisition of *Borrelia* spirochetes by *I. ricinus* nymphs. The results have been added to the Supplemental data (Fig S4), Material and Methods (now Lines 554-562), Results (now Lines 270-278), and Discussion (now Lines 446-452).

**Reviewer Figure 5.** The tick Toll pathway does not regulate acquisition of *Borrelia afzelii* by tick nymphs. (A) Efficacy of gene silencing in the fully-fed nymphs (whole bodies) measured by qRT-PCR. Each dot represents a single nymph. (B) Relative expression (qRT-PCR) of *defIR* in the dsRNA-injected, fully-fed nymphs. Each dot represents a pool of five nymphs. Gene expression in the dsGFP control was set as 1. (C) Absolute number (qRT-PCR) of *B. afzelii* in the fully-fed nymphs pre-injected with dsRNA analyzed 0, 1, and 2 weeks after detachment. *cac1* = *cactus1*, *dl* = *dorsal*, *defIR* = *defensin*. *P ≤ 0.05; **P ≤ 0.01; *n.s.* = not significant P ≥ 0.05.

**Reviewer #2**

Major Issue 1: Line 452: Please include information on how infections by these bacteria were verified. Also, were nymphs tested for the presence of these bacteria before the experimental infections were carried out? This should be something to take into account since previous exposure might affect their immune responses.

Our response to Major Issue 1: We have previously reported that ticks have a minimal bacterial load both before and after feeding, with the exception of endosymbionts in the ovary (DOI: 10.3389/fcimb.2020.00211). Most of these bacteria are intracellular species, mainly from the genus *Rickettsia*. To stimulate the tick immune system, we used established microbial species commonly used to characterize the immune system of *Drosophila* (DOI: 10.1038/nri219) and injected 32 nl of a freshly cultured microbial suspension (live microbes) into the tick hemolymph, which is considered a microbe-free environment in healthy ticks. One day after the injection, no lethal effects were observed in the ticks. Although it would be ideal to work with completely germ-free ticks, this poses a technical challenge. We did not feel this was necessary for our particular experiments, especially since we pooled a sufficient number of ticks per group, which likely minimized the potential for unbalanced results due to a higher bacterial load in individual ticks. The ticks we used for our experiments came from our tick rearing facility, where we regularly test for common tick transovarially-transmitted pathogens (e.g., *Babesia venatorum*) using PCR (Lines 408-409). Spirochetes such as *Borrelia burgdorferi* sensu lato and *Babesia microti* are generally not transmitted transovarially and the offspring of adult females that have fed on uninfected laboratory animals are considered free of these pathogens.

**Part III – Minor Issues: Editorial and Data Presentation Modifications**

**Reviewer #1**

Minor Issue 1: Fig 2C – was hard to see which treatment shows significant increase/decrease in DeflR transcripts – the asterisks appear to be on top of the M. luteus group (presumably it should be on the E. coli group).

Our response to Minor Issue 1: Thank you very much for this comment. The reviewer is probably referring to Fig 3C. We agree that the visualization in these graphs (Fig 3C, but also Fig 2A, 2E, and 3B) was not optimal. We have therefore revised the graphical representation of these images to improve them.

Minor Issue 2: There are two dorsal like proteins in Drosophila: DIF and Dorsal. One involved in development and one in immune response. I. scapularis has only one – and lacks the DIF (Dorsal-like immune factor) so also I. ricinus. The authors could provide some insights on this in the Discussion.

Our response to Minor Issue 2: The phylogenetic position of *Drosophila* Dorsal and DIF (Dorsal-related immunity factor) is shown in the phylogenetic tree in Fig 1. DIF appears to have evolved specifically in *Drosophila* and related species. In *Drosophila*, Dorsal plays a role in embryonic development, while DIF is specifically involved in adult immunity (DOI: 10.1038/nri2194). In ticks, DIF is absent and only Dorsal is present and fulfills both developmental and immune functions, as our results show. We now include this information in the Discussion (now Lines 359-362) as an interesting evolutionary aspect of the Toll pathway.

Minor Issue 3: The quantitative PCR results were normalized to ferritin for Babesia? For all other genes, results were normalized to elongation factor. An explanation for this would be helpful.

Our response to Minor Issue 3: We performed DNA-based relative quantification of *Babesia* in the salivary glands (and midgut) by qRT-PCR. DNA quantification was normalized against ferritin (*fer2*), although normalization can be performed with any tick DNA fragment to represent the relative number of tick genomes. For relative quantification of tick gene transcripts (RNA-based), we used elongation factor (*ef*), a commonly used housekeeping gene in our previous studies (DOI: 10.1016/j.dci.2014.09.004).

**Reviewer #2**

Minor Issue 1: Lines 409 and 444: add references to the methodology employed for PCR and qRT-PCR.

Line 478: Use the correct scientific notation, in this case: 5 x 10 to the 8.

References: Use italics for scientific names.

Our response to Minor Issue 1: Thank you for your feedback. We have made the necessary changes according to your comments.

**Reviewer #3**

Minor Issue 1: Line 31: Can vector competence be determined by the ability of pathogens to evade recognition? Would vector competence not focus on the vectors ability to acquire, maintain and transmit a pathogen to a host.This latter definition focus on the vector, but the former imply that the pathogen determine vector competence. What if parasite and vector co-evolved that the pathogen is not destroyed by the immune system. This is not evasion, but co-adaptation would increase vector competence. Perhaps rephrase the sentence?

Our response to Minor Issue 1: We greatly appreciate this comment and agree that the original wording of the first sentence in the Abstract may be misleading as it implies that the pathogen alone determines vector competence. To address this unintended implication, we have reworded the sentence to clarify that vector competence is influenced by a complex interplay of various vector-host interactions, including immunologic factors. The amended sentence in the Abstract: "The vector competence of blood-feeding arthropods is influenced by the interaction between pathogens and the immune system of the vector." (now Lines 31-32).

Minor Issue 2: L223, 231: Write out genus name when starting a sentence.

References

Check that species names are italicized and that the references follow the journal format.

Our response to Minor Issue 2: Thank you for your feedback. We have made the necessary changes according to your comments.
